# Exopolysaccharide production by lactic acid bacteria: the manipulation of environmental stresses for industrial applications

**DOI:** 10.3934/microbiol.2020027

**Published:** 2020-11-17

**Authors:** Phu-Tho Nguyen, Tho-Thi Nguyen, Duc-Cuong Bui, Phuoc-Toan Hong, Quoc-Khanh Hoang, Huu-Thanh Nguyen

**Affiliations:** 1 Graduate University of Sciences and Technology, Vietnam Academy of Science and Technology, Ha Noi, Vietnam; 2 Department of Biotechnology, An Giang University, An Giang, Vietnam; 3 Vietnam National University Ho Chi Minh City, Ho Chi Minh City, Vietnam; 4 Ho Chi Minh City University of Technology (HUTECH), Ho Chi Minh City, Vietnam; 5 Department of Pathology and Microbiology, University of Nebraska Medical Center, Omaha, NE, United States; 6 LAVI's Institute for Agricultural Science and Plant Breeding, Ho Chi Minh City, Vietnam; 7 Institute of Tropical Biology, Vietnam Academy of Science and Technology, Ho Chi Minh City, Vietnam

**Keywords:** exopolysaccharides, environmental stress, EPS biosynthesis, biofilm, EPS production

## Abstract

Exopolysaccharides (EPSs) are biological polymers secreted by microorganisms including Lactic acid bacteria (LAB) to cope with harsh environmental conditions. EPSs are one of the main components involved in the formation of extracellular biofilm matrix to protect microorganisms from adverse factors such as temperature, pH, antibiotics, host immune defenses, etc.. In this review, we discuss EPS biosynthesis; the role of EPSs in LAB stress tolerance; the impact of environmental stresses on EPS production and on the expression of genes involved in EPS synthesis. The evaluation results indicated that environmental stresses can alter EPS biosynthesis in LAB. For further studies, environmental stresses may be used to generate a new EPS type with high biological activity for industrial applications.

## 1. Introduction

In recent years, the trend of using natural polymers in many fields has led to the development of research on producing exopolysaccharides (EPSs) from bacteria. The unique structural features have made bacterial EPSs of particular interest in the fields of chemistry, medicine and food industry [1]. Because of their ability to increases hold water, EPSs are widely used as viscous, stabilizing and emulsifying agents in the food industry [2] to improve the rheological property, texture and sensibility of bread and fermented milk products such as yogurt and cheese [3]. In addition to its technological properties, EPSs also have potential health benefits as antioxidant, anticancer, anti-inflammatory antiviral activities [4],[5] and cholesterol lowering effects [6].

Among EPS producing bacteria, Lactic acid bacteria (LAB) have has grasped the attention of researchers thank to their strong ability to produce EPSs. The LAB strains as *Streptococcus, Lactococcus, Pediococcus, Lactobacillus, Leuconostoc* and *Weissellale* are often used to produce EPSs [7]. LAB are recognized as safe microorganisms (GRAS-Generally Recognized As Safe) and also capable of creating EPSs with many different structures without any health risks [8]. In LAB, EPSs play an important role in controlling cell surface physicochemical characteristics [9], protecting bacterial cells from dehydration, negative environmental impacts, antibiotics, phagocytosis, and phage attacks [10]–[12]. EPSs take part in the structural components of extracellular matrix, in which cells are encapsulated during the development of cell membrane [13].

Previously, there have been several reviews to describe the stress response in LAB [14]–[16] focusing almost exclusively on the function of stress proteins (HS proteins, Csp, etc.) and their regulators (HrcA, CtsR) or that of proteins linked physically to the cell membrane (transport systems, sensors, housekeeping proteases, etc.). However, in order to understand clearly the role of stresses in EPS biosynthesis, a series of key questions must be addressed:

-Why are EPSs related to stress resistance?

-What type of stress to apply?

-Is it possible to control EPS biosynthesis using environmental stress?

Therefore, in this review, we will discuss EPS synthesis; the physiological functions of EPSs as well as the impact of environmental stresses on EPS production and the expression of genes involved in EPS biosynthesis in LAB. This assessment will clarify the relationship between environmental stresses and changes in LAB EPS synthesis. It also suggests that environmental stress can improve the productivity of EPSs from LAB and produce customized EPSs with desired functionality.

## 2. EPSs biosynthesis in LAB

**Figure 1.**
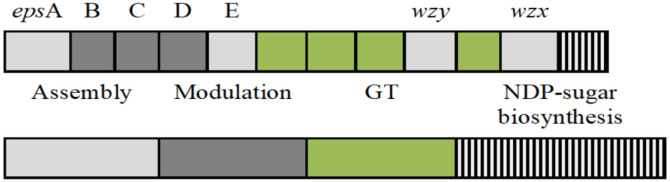
Schematic genetic organization of the *eps* gene clusters [20].

LAB synthesize two types of EPSs including homopolysaccharides and heteropolysaccharides [17]. Homopolysaccharide synthesis is a relatively simple biochemical process involving a specific GT (glucansucrase or fructansucrase) and an extracellular sugar donor (sucrose for the synthesis of glucans, but it can also be other fructose-containing oligosaccharide (e.g. raffinose) for the synthesis of fructans) [18],[19]. Heteropolysaccharide synthesis is a complex process which involves the specific role of several gene products (enzymes) encoded by the *eps* gene cluster and housekeeping genes. These gene products can be categorized into four groups (or modules) basing on their functions: polysaccharide assembly machinery (the priming glycosyltranferases, Wzx or flippase, Wzy or polymerase and EpsA), phosphoregulatory system managing polysaccharide assembly (EpsB, EpsC and EpsD), glycosyltranferases and sugar nucleotide biosynthetic pathways. Genes encoding acetyl- and pyruvyl transferase involved in the chemical decoration of EPSs also present in the cluster (Figure 1) [20].

**Figure 2.**
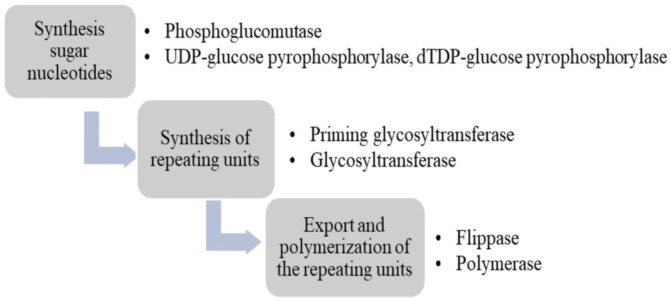
The steps of biosynthesis of EPS and related enzymes.

In general, EPS biosynthesis is summarized in 3 main steps (Figure 2). Firstly, it is the generation of activated sugar precursors (or sugar nucleotides such as uridine diphosphate glucose and thymidine diphosphate glucose) for repeating units. The sugar nucleotides are synthesized in multistep pathways from glycolytic intermediates, generally glucose-6-phosphate or fructose-6-phosphate. This complex process requires the function of several housekeeping gene products such as phosphoglucomutase (converts glucose-6-phosphate to glucose-1-phosphate) [21]; UDP-glucose pyrophosphorylase and dTDP-glucose pyrophosphorylase (converts glucose-1-phosphate to sugar nucleotides UDP-glucose and dTDP-glucose, respectively) [22]. The producing potential of different sugar nucleotides is intrinsically determined by the gene content of each LAB, which ultimately dictates the type of monomers found in EPSs. EPSs produced by LAB consist of repeating units which is usually composed of two or more (usually 3–8) types of monosaccharides [22]–[24].

Secondly, the synthesis process of repeating units begins by attaching the first sugar nucleotide to the isoprenoid lipid carrier, undecaprenyl phosphate, which is attached to the cytoplasmic membrane of the cell, and being catalyzed by priming glycosyltransferase. This is followed by the sequential addition of sugar nucleotides to form repeating units and glycosyltransferases encoded by gense in the *eps* gene clusters catalyze for this process [25]. Finally, it is the polymerization and export of repeating units from the inner part to the outer part of cell membrane. Basically, three different proteins which are also encoded in the *eps* gene cluster carry out polymerization and export process: A flippase (encoded by *wzx* or *cps*J) or a translocase moves the lipid carrier-repeating unit complex from the inner surface of cytoplasm membrane to periplasmic. Then, a polymerase (encoded by *wzy* or *cps*H) catalyzes the coupling of repeating units [21]. Lastly, a chain length determination protein separates lipid carrier-repeating unit complex to stop polymerization and export process simultaneously determines the chain length of final EPSs [21].

EPSs are synthesized to serve various functions in the bacteria. One of these is to ensure bacteria survive under stress conditions. The function of EPSs in LAB's stress resistance is discussed in the next section.

## 3. The role of EPSs for LAB stress resistance

EPSs are the important structural component of LAB cell wall [26]. EPSs form a layer surrounding cells to protect them against adverse environmental conditions such as dehydration, extreme temperature, acid, osmotic stress, phagocytosis, macrophages, and antibiotics [25],[27],[28]. Other roles of EPSs include biofilm formation, cell adhesion mechanisms [29] and the determinant of strain-specific characteristics in host interaction [30].

**Figure 3.**
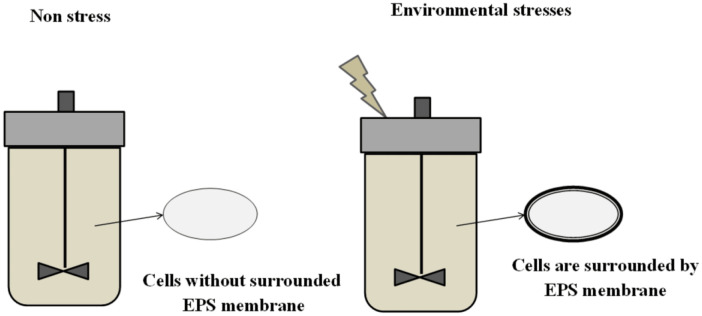
Fermented system for studying roles of environmental factors on EPS production.

To adapt to environmental stresses, LAB can alter their cell surface by producing more EPSs [31]. The increased production of EPSs results in thicker and firmer cell walls (Figure 3). As a result, it increases the LAB's resistance to stresses. This feature may be useful to exploit for improving the stamina of the probiotic starters as well as the ability to produce EPSs in LAB. Numerous studies have also demonstrated that, after being pre-stressed, LAB's viability is improved significantly [31],[32].

**Figure 4.**
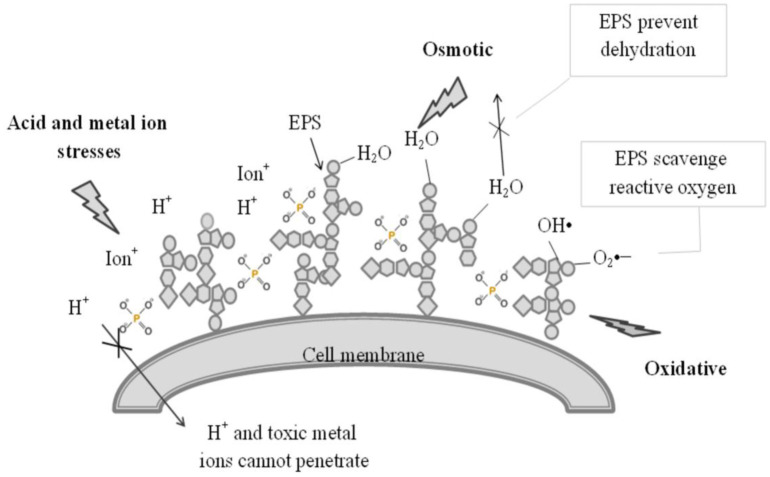
The role of EPSs in acid, osmotic, oxidative stress resistance.

As discussed, LAB enhance EPSs synthesis to create a physical barrier on the cell surface which separates the cell from stress. Especially, in low pH conditions, this EPS layer restricts the access of exogenous acids to bacterial cells due to the anions bound to EPSs as phosphate groups [33]. Phosphate residues confer a net negative charge to EPSs [27]. The presence of phosphate in EPSs is also observed in many studies [34]–[37]. According to these viewpoints, LAB may produce anionic EPSs carrying phosphate groups under acid stress conditions and they cause negative charge on cell surface to prevent proton diffusion into cells (Figure 4).

In the case of osmotic stress, a sudden increase in osmotic pressure made by stress results in water movement from the inside to the outside of cell, causing a detrimental loss of cell turgor pressure and changing intracellular solute concentration, which ultimately can seriously affect cell viability [38]. In response to osmotic stress, LAB synthesize EPSs to protect themselves by holding water around cells to prevent dehydration (Figure 4) [39]. The water holding capacity of EPSs is due to the presence of OH groups in their structure. Another substance such as glycerol known for high water holding capacity can sometimes be included in the structure of EPSs. The presence of glycerol was recorded in EPSs produced by *Latilactobacillus sakei* [34]. Furthermore, external protective compounds such as water stress proteins which aid in the survival of cells from desiccation can accumulate in extracellular glycan and show homologies with carbohydrate-modifying enzymes [40].

A lot of bacteria respond to carbon dioxide stress by producing EPSs to create a barrier for slowing the diffusion of toxic substance into cells, which in this case would be carbon dioxide [41],[42]. Similarly, EPSs also provides support for LAB to resist metal stress. The negatively charged groups in EPSs bind cations and protect bacterial cells against toxic metals [43]. In addition, it has been shown that EPSs are related to oxidative stress resistance in LAB. The supplementation of EPSs into culture medium could appreciate the growth of *L. mesenteroides* by 10 times under oxidative stress and influence promoting the aerobic growth of oxygen-sensitive strains such as *Lactobacillus* and *Bifidobacterium* [44]. In oxidative stress conditions, the production of harmful reactive oxygen species may be increased. EPSs can scavenge of these reactive oxygen species to prevent cell damage (Figure 4) [45]. Furthermore, EPSs also reduce oxidative stress by extrusion of dissolved oxygen from aqueous culture medium [44].

The ability to protect cells from environmental stresses depends on EPS-phenotype. Terms such as ‘ropy’, ‘mucoid’, and ‘slime’ have been used to describe the different EPS producing phenotypes of LAB [46]. LAB strains with the ropy-exopolysaccharide production show better resistance to stress. According to a report, the ropy phenotype of *Lactiplantibacillus plantarum* is related to better tolerance to low pH [29].

## 4. The physiological functions of EPSs

Together with the cell protection function, the positive advantages of EPSs are highlighted through the essential contribution to the human health such as prebiotic, anticoagulant, antioxidant, anti-inflammatory, antiviral, cholesterol lowering effects and even anticancer activity [47].

LAB's EPSs have been showed an essential functional role in blood coagulation prevention. The strong anticoagulant activity of EPSs in sulphate derivatives has been demonstrated. Heparin Cofactor II is a potent inhibitor of thrombin in the coagulation pathway and the sulphated EPSs provides an acidic medium condittion to facilitate the inhibitory effect of Heparin Cofactor II on thrombin [48],[49]. The sulphated sites and stereochemistry of EPSs activate HC II according to the allosteric mechanism [50]. One study proved that EPS47FE and EPS68FE which are secreted by *L. plantarum* 47FE and *Lactiplantibacillus pentosus* 68FE, respectively, exhibit strong anticoagulant and fibrinolytic activity [51].

Prebiotic effects were also observed at LAB's EPSs [52],[53]. EPSs from LAB can be used by probiotic strains [54] and have the capacity to stimulate the growth of probiotic bacteria and maintain the balance of intestinal microflora [55],[56]. The prebiotic potential of LAB's EPSs has demonstrated in many studies. The α-D-glucan synthesized by *L. plantarum* can stimulate probiotic bacteria growth. It is low-digested by artificial gastric juice and show to put non-probiotic bacteria off growing that *Enterobacteriaceae* is a representative instance [57]. *In vitro* EPSs produced by *Weissella cibaria*, *Weissella confusa*, *L. plantarum* and *Pediococcus pentosaceus* could be used as a prebiotic ingredient in the food industry to modulate gut microbiota towards health benefits [58].

Another health promoting functions of EPSs produced by LAB are cholesterol lowering effects [50]. In an *in vitro* assay, EPSs produced by *L. plantarum* BR2 show cholesterol lowering properties (45%) [59]. Based on animal and *in vitro* experiments, several hypotheses to explain the cholesterol lowering mechanism of EPSs have been proposed including bile removal, anabolism and cholesterol conversion, co-precipitation effects, etc...[60],[61].

Free radicals usually cause serious health problems. Therefore, EPSs are such an important natural antioxidant to prevent the free radicals. LAB's EPSs also exhibit high antioxidant activity. One evidence showed that EPSs from *Lactobacillus gasseri* FR4 have a good free radical activity, while hydroxyl and superoxide radical capture activities are dependent on EPS concentration [62]. Additionally, under *in-vivo* conditions, LAB'S EPSs have been shown to increase the activity of hepatic superoxide effutase, serum catalase, and glutathione S-transferase simutaneously reducing serum malondialdehydes and monoamine oxide activity. These are excellent antioxidant and anti-aging evidence created by EPSs [63],[64].

In recent decades, the immunomodulatory potential of EPSs has received a lots of scientific consideration. Many in-vitro studies have demontrated that EPSs produced by different LAB species have the immunomodulatory ability [17]. The phosphate group (a good inducer of the immune response) plays a critical role and characterizes the immunomodulatory effects of EPSs. Phosphate molecules can activate various immune cells (such as macro-phages and lymphocytes) and initiate immune responses [65]. According to these results, it can be speculated that EPS generated under acid stress (it seems that acidic EPSs [65]) may exhibit stronger immunological properties.

Cancer is one of the health problems getting a lot of attention today and it is usually treated via chemotherapy method. However, chemotherapy can cause some unexpected effects which can range from minor to severe and life-threatening [66]. Therefore, other pharmaceutical products are being researched to help cure cancer that LAB's EPSs are the helpful ones due to its anti-tumor effects [65]. EPSs from *L. plantarum* 70810 can significantly inhibit the proliferation of tumor cells such as HepG-2, BGC-823, especially HT-29 [67]. *In vitro* evaluation of anticancer properties of *Lactobacillus acidophilus* EPSs in colon cancer cell lines demontrated that they were able to inhibit the expression of genes involved in angiogenesis and tumor survival [68]. In another study, EPSs from *Levilactobacillus brevis* MSR10 are used to synthesize the silver nanoparticles (AgNPs). According to the results obtained, these AgNPs not only had high antimicrobial and antioxidant capabilities but also significantly reduced the percentage of live HT-29 cells [69].

Many recent researches have been conducted to show the antiviral bioavailability of EPSs and they are considered to be an immune stimulant affect in a number of ways in the immune system, contributing to the protection of human cells against certain viruses [65]. A study has proven that EPSs extracted from *L. plantarum* LRCC5310 were able to resist human rotavirus in vitro [70].

## 5. Impact of environmental stresses on EPS production in LAB

Under environmental stresses, LAB have different adaptation mechanisms which involve the accumulation of compatible solutes and energy storage compounds; regulation of energy production pathways, as well as the modulation of cell envelope, i.e., membrane, cell wall, surface layers, and EPSs [71]. In this review, we divide environmental stresses into common groups including nutrient factors (carbon sources, nitrogen source, carbon dioxide, oxigen, mineral salts, etc.); physiological factors (pH, osmotic stress, temperature, etc.) and co-cultivation to discuss the impact of stresses on EPS production in LAB.

### 5.1. Nutrient stress factors

The composition of nutrients is one of the factors which affects the growth and metabolism of cells [72]. Thus, EPS synthesis is also influenced by culture medium compositions [73]. The starvation or oversupply of nutrients such as nitrogen, sugars, carbon dioxide, etc. may change EPS synthesis [74]–[76]. Effects of nutritional stress on EPS synthesis in LAB have been proved by prior studies. Marshall *et al*. demonstrated that EPS production in *Lactobacillus lactis* subsp. *cremoris* LC 330 is stimulated by nitrogen limitation [77]. In contrast, *Lactobacillus delbrueckii* ssp. *bulgaricus* was recorded increased EPS production in additional nitrogen-enriched [78].

Excessive sugar presence in the culture medium also increases EPS production in LAB. The possible explanations for the increased EPS synthesis under stress of high sugar concentration are osmosis, unlimited supply of sugar building blocks and high energy availability [75]. The increased sucrose concentration in the MRS medium was suitable for EPS overproduction in *Lactobacillus confusus* TISTR 1498 [79]. It has also been showed that *Lactobacillus* strains (*L. delbrueckii bulgaricus*, *Lactobacillus helveticus* and *Lacticaseibacillus casei*) yield the highest EPSs when growing on fermentation medium comprising 20% sucrose as carbon source [80]. Likewise, the synthesis of EPSs in *Fructilactobacillus sanfranciscensis* LTH2590 rose by increasing the sucrose concentration in the medium and reached about 40 g/L at sucrose concentration of 160 g/L [81]. In the case of *Leuconostoc mesenteroides* NRRL B-1299, culture medium with sucrose concentration over 5 g/L caused more dextran production [82]. Similar to succorse, the high concentration of glucose is also advantageous for the production of EPSs. As previously reports, the EPS production of *Streptococcus thermophilus* (W22) and *L. delbrueckii* subsp. *bulgaricus* (B3, G12) was stimulated by high glucose concentration [83]. It was also shown that the presence of excess sugar in medium has a improving effect on EPS production in *L. casei* and *Lacticaseibacillus rhamnosus*, although the growth is apparently decreased [84],[85].

In some LAB strains, carbon dioxide can used as a carbon source for growth because it is a substrate in carbamoyl phosphate synthesis and other metabolic reactions in LAB [86]. Carbon dioxide regulates physiology and energy metabolism by regulating enzymes involved in glycolysis [87]. The impact of carbon dioxide stress on EPS production in LAB has also recorded in several studies. EPS production depended entirely on carbon dioxide concentration and the maximum EPS yield, produced by *Bifidobacterium longum* JBL05, increased proportionally to carbon dioxide concentration in the range of 0–20% [88]. *L. casei* growing in carbon dioxide-rich environment was surrounded by a membrane like EPS component [89]. In contrast to carbon dioxide stress, under dissolved oxygen concentration above 0.05 ppm, *B. longum* declined growth and EPS accumulation [90]. These results suggest that the EPS synthesis of *B. longum* varies under different stress conditions. Although oxidative stress reduces the accumulation of EPSs in *B. longum*, it increases EPS production in *B. scardovii* and *B. adolescentis*. One evidence has shown an increase in EPS production and the cell surface hydrophobicity of *B. scardovii* and *B. adolescentis* under oxidative stress [91].

### 5.2. Physical stress factors

EPS production in LAB may be stimulated by various physical stresses as a cellular defense response, which could also enhance the formation of biofilms [92]. The rate of EPSs in biofilms can account for about 50–90% of total organic matter amount [93],[94] and EPSs, together with proteins, nucleic acids and lipids, form the structure of a biofilm matrix [95]. Low pH was found to significantly decrease the formation of biofilm in *L. rhamnosus* GG, while it enhanced biofilm formation in *Limosilactobacillus reuteri* strains [96],[97]. Although studies have not focused on the effect of low pH stress on EPS production in LAB bacteria, an increase in EPS production under low pH has been observed in several reports. The EPS production of *Lactobacillus helveticus* ATCC 15807 under controlled pH of 6.2 was lower than that observed at pH 4.5 [98]. Likewise, EPS production in *Ligilactobacillus salivarius* UCO_979C-2, adapted variant strain, after 24 h at pH 2.6 was 690 mg/L, compared to native *L. salivarius* UCO_979C-1 strain that was only 450 mg/L at pH 6.4 [99].

The negative effects of osmotic stress on cells may be limited because of the presence of EPSs. Therefore, the presence of substances caused high osmotic pressures, such as NaCl, can stimulate EPS synthesis on the cell wall. As previously described by Seesuriyachan *et al*., the EPS synthesis of *L. confusus* TISTR 1498 did not depend on biomass and stress of high NaCl concentration could enhance EPS production in solid state fermentation [79]. Similarly, *Leuconostoc mesenteroides/pseudomesenteroides* 406 achieved maximum EPS yield in the presence of 5% NaCl [100]. In contrast, the inhibition of EPS production by NaCl was recorded in *L. helveticus* ATCC 15807 [98].

Excessive temperature causes protein denaturation, nucleic acid and membrane damage [101]. However, when bacteria are exposed to extreme temperature, they reprogram their metabolism to deal with temperature changes [102]. One of the metabolic changes is an increase in EPS synthesis. High temperature stress is also recorded to affect EPS production in LAB. Nguyen *et al*. demonstrated that sub-lethal thermal stress increases EPS production and improves the viability of *B. bifidum* [31].

### 5.3. Co-cultivation

In biotechnology, co-culture has been shown to make microorganisms more resistant to environmental changes and can perform more complex metabolic activities through the culture combination of various strains [103],[104]. Consequently, co-culture can also affect EPS synthesis. The effect of co-cultivation on improving EPS production of LAB is often studied in combination with *Saccharomyces cerevisiae*. *Lactobacillus kefiranofaciens* JCM 6985 enhanced the production of kefiran, an exopolysaccharide, in co-culture with *S. cerevisiae* IFO 0216 [105]. The EPS production of *L. rhamnosus* strains was also increased by 39–42% and a higher level of EPS operon expression was observed for *L. rhamnosus* RW-9595M in co-culture [106]. Similarly, *L. paracasei* co-cultured with *Saccharomyces cerevisiae* resulted in the overexpression of gene (coding for polyprenyl glycosylphosphotransferase) involved in EPS production [107]. In facts, the enhancement of EPS production by LAB in co-culture with *Saccharomyces cerevisiae* is induced by direct and physical contact with components on the surface of yeast cell [105]. In a high viscosity environment, LAB can be stressed by themselves own acids. LAB adhesion to yeast cell will activate EPS production in LAB because this adhesion leads to efficient lactic acid consumption by yeast cells [105].

In general, the biosynthesis of EPS can be altered either up or down under different stress conditions. These changes may be related to expression level of genes involved in EPS synthesis. To clarify this hypothesis, we have discussed the expression of *esp* genes under environmental stresses. Details are presented in section 6.

## 6. Changes the expression of genes involved in EPS production under environmental stresses

**Figure 5.**
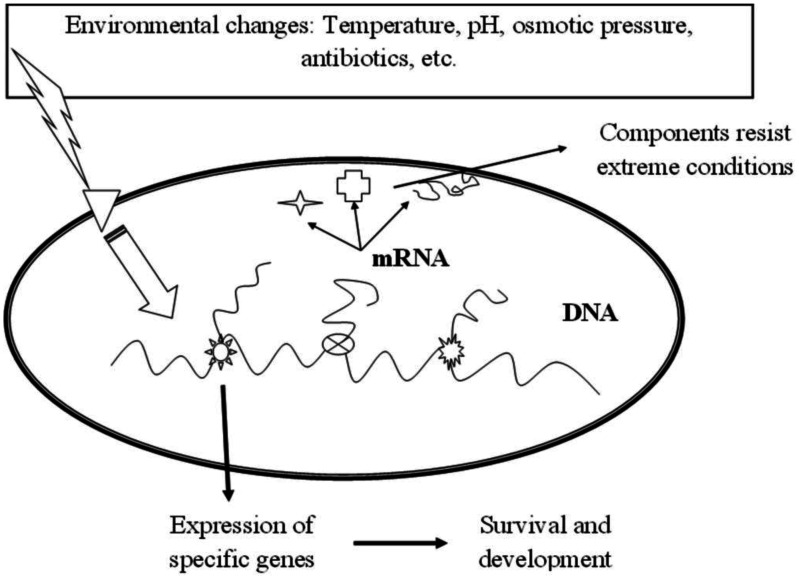
The mechanism of transcription regulation against environmental stress in LAB.

Bacteria respond to stresses by activating various regulatory mechanisms including activities involved in metabolisms, cell envelope and gene expression, giving them the potentiality to adapt to extreme environmental conditions (Figure 5) [108]. Changes in gene expression establish the principal component of the bacterial response [109] and can alter the biosynthesis of EPSs under stress conditions [110].

The correlation between stress and the expression of genes involved in EPS biosynthesis has been documented in LAB. Increasing expression of *gtf*01207 gene, encoding for a priming glycosyltransferase related to EPS synthesis, was observed in *B. animalis* subsp. *Lactis* after exposure to stress of acid, bile salts and osmosis [92],[111]. According to another study, when the pH of culture medium decreased from pH 6.5 to pH 5.5, the expression level of *eps*NMLKJ genes in *Streptococus thermophilus* ASCC 1275 increased. However, the expression of genes involved in the synthesis of sugar nucleotides such as dTDP-rhamnose and UDP-GlcNAc reduced [112]. Also in this study, when temperature increases from 37 °C to 40 °C, there are not changes in the expression level of *eps*NMLKJ cluster, but the expression of *eps*1C and *eps*1D genes increase while that of *eps*2C and *eps*2D decrease [112]. Expression of *gtf* gene encoding for enzyme which produces beta-glucan (membrane-linked glycosyltransferase enzyme) in *Lacticaseibacillus paracasei* caused 60 times higher heat tolerance, 20 times higher acid tolerance compare to control strain [113]. Evaluation of gene expression is not only based on mARN but also on genetic products which are enzymes formed after decoding. Glyceraldehyd-3-phosphate dehydrogenase, proved to be necessary for EPS production of *Xanthomonas campestris pv*. [81], increased heterological expression in *L. rhamnosus* HN001 during heat stress. In contrast, the heterological expression levels of glyceraldehyd-3-phosphate dehydrogenase and phosphoglycerate kinase, related to EPS biosynthesis of *Xanthomonas axonopodis pv*. Glycines) [114], decreased under osmotic stress [115]. In general, environmental stress can alter the expression of genes involved in EPS biosynthesis. The result of this response may increase EPS production in LAB.

## 7. Conclusions and future trends

In order to survive under environmental stress conditions, LAB react by synthesizing EPSs to form a protective barrier around the cells. This EPS synthesis is catalyzed by enzymes encoded by genes in the *eps* cluster and the impact of environmental stresses can alter the expression of these genes resulting in increased EPS production. Accordingly, environmental stress may be considered as a major factor to control LAB's EPS biosynthesis. The impact of different stresses on EPS synthesis is summarized in Table 1. In general, the synthesis of EPSs in LAB under stress conditions depends on the type of stress and bacterial species. Within the same species, EPS production may not be the same under different stress conditions. For instance, EPS production in *L. helveticus* ATCC 15807 is stimulated by stress at low pH but inhibited by sodium chloride stress. Conversely, a specific stress may stimulate EPS production in one species but inhibit it in another (Table 1).

**Table 1.** Impact of stress conditions on LAB EPS synthesis.

Stress exposure	Strains	Effect on EPS production: Inhibition (-) Stimulation (+)	References
Low pH	*L. rhamnosus* GG	−	Lebeer *et al.*, 2007 [97]
	*L. reuteri*	+	Slížová *et al*., 2015 [98]
	*L. helveticus* ATCC 15807	+	Torino *et al*., 2005 [99]
	*L. salivarius* UCO_979C-2	+	Sanhueza *et al*., 2015 [100]
High temperature	*B. bifidum*	+	Nguyen *et al*., 2014 [31]
Sodium chloride	*L. helveticus* ATCC 15807	−	Torino *et al*., (2005) [99]
	*Leuconostoc mesenteroides/pseudomesenteroides* 406	+	Silvia-Simona GROSU-TUDOR, 2014 [101]
	*L. confusus* TISTR 1498	+	Seesuriyachan, 2012 [80]
Carbon dioxide	*B. longum* JBL05	+	Ninomiya *et al*., 2009 [89]
	*L. casei*	+	Santillan *et al*., 2015 [90]
Oxidation	*B. longum*	−	Golowczyc *et al*., 2011 [91]
	*B. scardovii; B. adolescentis*	+	Qian, Borowski, & Calhoon, 2011 [92]
Excessive nitrogen source	*L. delbrueckii* ssp. *bulgaricus*	+	García-Garibay & Marshall, 2008 [78]
Excessive carbon source	*L. confusus* TISTR 1498	+	Seesuriyachan *et al*., 2012 [80]
	*L*. (*delbrueckii bulgaricus*, *helveticus* and *casei)*	+	Hussein *et al*., 2015 [81]
	*F. sanfranciscensis* LTH2590	+	Korakli, Pavlovic, & Vogel, 2003 [82]
	*Leuconostoc mesenteroides* NRRL B-1299	+	Dols, Remaud-Simeon, & Monsan, 1997 [83]
	*S. thermophilus* W22 and *L. delbrueckii* subsp. *bulgaricus* (B3, G12)	+	Yuksekdag & Aslim, 2008 [84]
	*L. Casei* CG11	+	Cerning *et al*., 1994 [85]
	*L. rhamnosus C83*	+	Gamar, Blondeau, & Simonet, 2003 [86]
Co-cultivation	*L. kefiranofaciens* JCM 6985	+	Tada *et al*., 2007 [106]
	*L. rhamnosus (ATCC 9595, R0011, and RW-9595M)*	+	Bertsch, Roy, & LaPointe, 2019 [107]
	*L. paracasei* ATCC 334	+	Yamasaki-Yashiki, Sawada, Kino-oka, & Katakura, 2016 [108]

The EPSs produced by LAB can be the key ingredients showing promising functional roles for various utilities in food, medicine, etc. However, low EPS productivity could be a problem limiting commercial applications of these EPSs. Currently, EPS production improvement studies often focus on optimizing culture mediums, using genetic engineering, using cheap fermentation substrates, and environmental stress [116]. As discussed, EPSs protect LAB from negative environmental effects. Consequently, environmental stresses can promote EPS synthesis in LAB. This feature can be useful to exploit to improve the stamina of probiotic starters and the yield of EPSs.

In addition, the biological activities of EPSs such as prebiotic, anti-oxidant, anti-inflammatory, ... are related to the monosaccharide compositions of EPSs. It has been proved that EPSs with distinct monosaccharide compositions vary in their therapeutic effects [117]. For instance, the proportion of monosaccharides (galactose > rhamnose > glucose) in the composition of EPSs produced by *L. reuteri* Mh-001 was demonstrated to relate to their anti-inflammatory activity, in particular galactose content enhances EPS anti-inflammatory effects on the macrophages [118]. Similarly, rhamnose-containing EPSs have been used in cosmetic applications because of owning to their emulsifying activity [119]. For further studies, we believe that environmental stresses may be an effective method which positively alters EPS biosynthesis to generate a new EPS type with higher biological activity for industrial applications.
